# Tyrosine kinase inhibitor sensitive PDGFRΑ mutations in GIST: Two cases and review of the literature

**DOI:** 10.18632/oncotarget.22663

**Published:** 2017-11-26

**Authors:** Pieter A. Boonstra, Jourik A. Gietema, Albert J.H. Suurmeijer, Matthew R. Groves, Fernando de Assis Batista, Ed Schuuring, Anna K.L. Reyners

**Affiliations:** ^1^ University of Groningen, University Medical Center Groningen, Department of Medical Oncology, Hanzeplein, Groningen, The Netherlands; ^2^ University of Groningen, University Medical Center Groningen, Department of Pathology, Hanzeplein, Groningen, The Netherlands; ^3^ University of Groningen, Faculty of Science and Engineering, Antonius Deusinglaan, Groningen, The Netherlands

**Keywords:** GIST, PDGFRα mutation, circulating tumor DNA, ddPCR, non-D842V

## Abstract

Gastrointestinal stromal tumors (GISTs) are rare mesenchymal malignancies of the gastrointestinal tract. Most GISTs harbor a c-KIT (80%) or a PDGFRα (10%) mutation that leads to constitutive activation of the tyrosine kinase receptor. Response to treatment with tyrosine kinase inhibitors (TKIs) is dependent on mutational status of the tumor. The most common mutation in PDGFRα, D842V, is known to be imatinib resistant. Almost all other PDGFRα mutations are imatinib sensitive. We describe two patients with a PDGFRα exon 18 mutated GIST responding to treatment with TKIs. One of these patients has a p.M844_S847 deletion, not previously described in relation with TKI treatment response. Mutations in circulating tumor DNA were detectable with digital droplet PCR in serial plasma samples taken during treatment and correlated with treatment response of both patients. Computer 3D-modeling of the PDGFRα kinase domain of these two variants revealed no direct interference in imatinib or sunitinib binding and no effect in its activity in contrast to the reported structure of the imatinib resistant D842V mutation.

An overview is given of the literature regarding the evidence of patients with different PDGFRα mutated GISTs on response to TKIs. The findings emphasize the use of mutational analysis in GIST to provide patients personalized treatment. Detection of mutations in plasma is feasible and can provide real-time information concerning treatment response. We suggest to register GIST patients with these uncommon mutations in a prospective international database to understand the tumor biology and obtain more evidence of such mutations to predict treatment response.

## INTRODUCTION

Gastrointestinal stromal tumors (GISTs) are rare mesenchymal malignancies of the gastrointestinal tract with an incidence of 10 cases per million people [1]. About 50% of GIST arises in the stomach, 30% in the small intestine and 20% in other parts of the gastrointestinal tract [2]. Tumors originate from the interstitial cells of Cajal (or its precursor cells), the smooth muscle pacemaker cells. Constitutively activating mutations in the genes coding for the tyrosine kinase receptors KIT or platelet derived growth factor alpha (PDGFRα) play a crucial role in the biology of these tumors [3]. Approximately 80% of GIST harbor mutations in KIT, 10% in PDGFRα. The remaining part are wild type, has a BRAF mutation or inactivation of the SDH complex. KIT exon 11 mutant tumors can occur anywhere in the gastrointestinal tract, whereas PDGFRα-mutant tumors arise primarily in the stomach, mesentery and omentum. KIT exon 9 mutant tumors are mostly found in the small intestine [4].

Surgery is the only curative treatment and treatment of choice when feasible. Patients with irresectable tumors due to local advancement or metastatic disease can be treated with imatinib mesylate, a KIT selective tyrosine kinase inhibitor (TKI) in neo-adjuvant and palliative setting. Response on systemic treatment is strongly dependent on mutational status of the tumor. Patients with an imatinib-sensitive mutation have a response or stable disease for a median time of 27 months [5]. When imatinib treatment fails, second line treatment with sunitinib and third line treatment with regorafenib is available [6]. Resistance in patients who have an imatinib sensitive primary mutation occurs often as a result of secondary mutations in the tumor that develop during treatment [7]. After potentially curative surgery, patients with PDGFRα mutations and those with wild-type GIST have a lower risk of recurrence than patients with KIT mutations [8]. Once recurrences occur, the most common PDGFRα mutation in exon 18 (D842V) is known to be resistant to imatinib treatment. But not all GISTs with a mutation in exon 18 of the PDGFRα gene are resistant to treatment with a TKI. Since the introduction of mutation analysis in biopsies of GIST tumors, it is known that specific PDGFRα mutations appear to be imatinib sensitive [9]. Response to therapy is generally evaluated by radiological imaging. Recent advances in molecular biology enable the detection of tumor mutations in circulating tumor DNA (ctDNA) in plasma. This plasma mutational load can reflect the treatment response and current disease state [10].

Two patients with various PDGFRα deletions who responded on TKI treatment are described and serial plasma samples of both patients were analyzed with digital droplet PCR (ddPCR). Finally, an overview of literature concerning PDGFRα mutations in GIST is presented.

### Clinical summary patient 1

**Figure 1:**
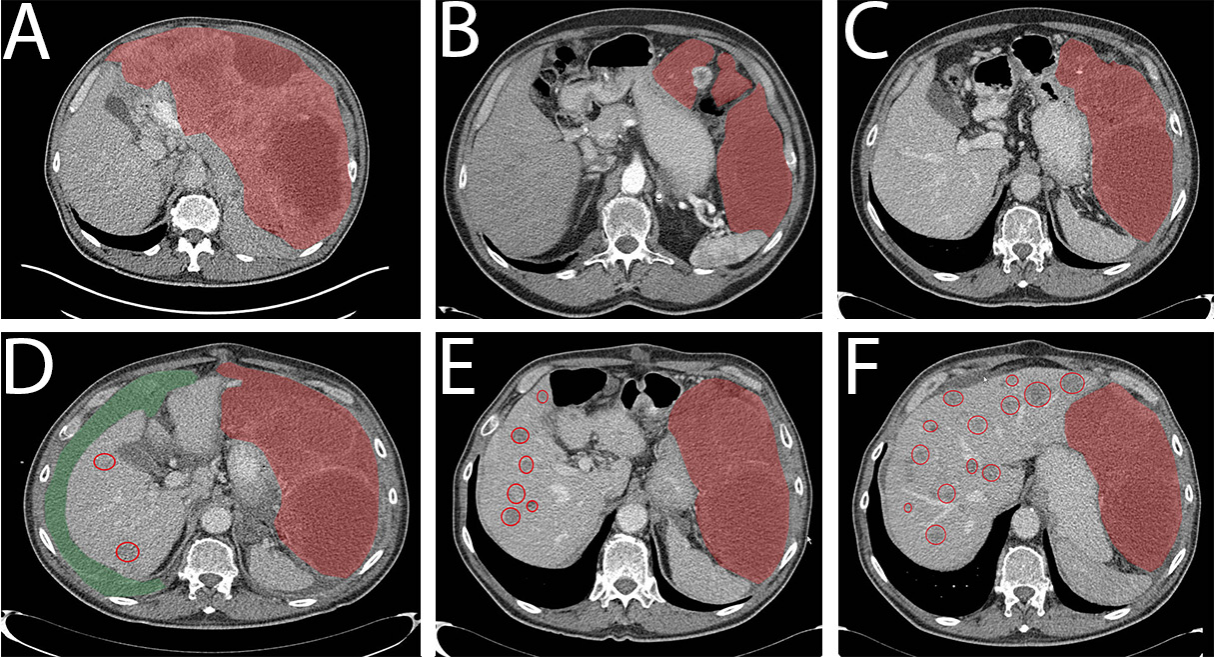
CT images of patient 1 Primary GIST (red area), liver metastases (red circles) and ascites (green) are indicated. **A.** Pre-treatment scan. **B.** After 12 months imatinib treatment, growing nodule. Start sunitinib. **C.** 6 months on sunitinib, stable disease. **D.** One year sunitinib. Progression. Ascites, liver metastasis. Start regorafenib. **E.** 3 months regorafenib, stable disease. Demarquation of the liver metastasis is seen as response on therapy. **F.** 6 months regorafenib, progression, more liver metastases are seen.

A 76-year old man was referred with a large abdominal tumor suspected for GIST. His WHO-performance score at first presentation was 3, being bed bound for the majority of the day. CT and FDG-PET scan showed a 29 cm large, irresectable tumor without evidence of metastases (Figure 1A). Mutation analysis on the performed biopsy specimen confirmed the diagnosis GIST with a mutation in exon 18 of PDGFRα (NM_006206.5: c.2531_2542del; p.(M844-S847del)). Treatment with imatinib 400 mg once daily was initiated in a neo-adjuvant setting. After one week he reported a clinical relevant benefit by disappearance of nausea and increasing energy levels. On the CT-scans performed every three months, stable disease was seen during one year of treatment. Based on a growing nodule (Figure 1B), progression was suspected and surgical resection of the tumor was considered. However, progression with peritoneal metastasis was seen on the following CT scan three months thereafter. Surgery with curative intent was no longer feasible and 15 months after start of imatinib, treatment was switched to second line treatment with sunitinib 37.5mg daily. The patient responded during one year on sunitinib (Figure 1C), until he was admitted to the hospital with malaise and ascites based on disease progression (Figure 1D). He recovered after drainage of ascites. A biopsy of a progressive nodule was performed and treatment with regorafenib (160mg daily for 3 out of 4 weeks) was started. With this regimen stable disease during 5 months was obtained (Figure 1E). He died one month after stopping treatment with regorafenib due to progressive disease (Figure 1F), no clinical benefit was reached with a re-challenge of imatinib. Plasma samples were available during treatment with regorafenib and 4^th^ line imatinib. An increase in mutational copies/ml is seen between stable disease (Figure 1E) and progression (Figure 1F) of 180 to 850 mutant copies/ml (Figure 3A). At the last visit to the outpatient clinic (two weeks before death), mutant copies were 4767 copies/ml (month 33).

**Figure 2:**
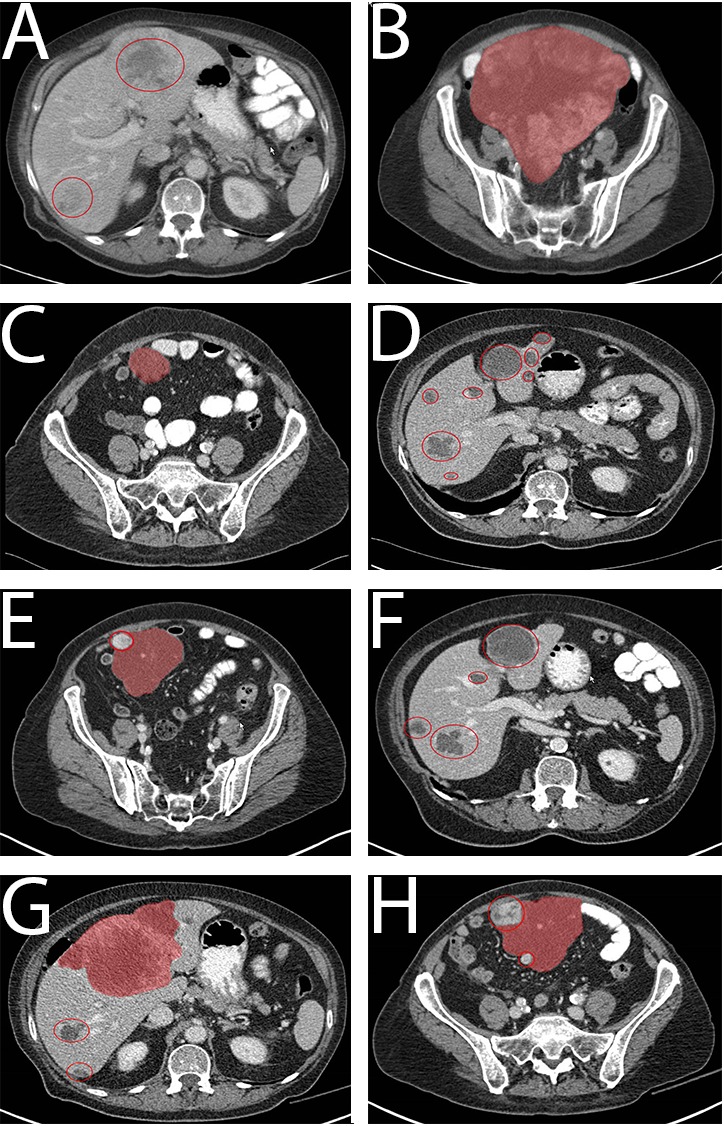
CT images of patient 2, left column images of pelvis, right column images of liver at the same time Primary tumor (red area), metastases (red circles). **A.** Pretreatment, large tumor in the lower abdomen. **B.** Pretreatment, multiple liver metastases. **C.** 24 months imatinib, treatment response. **D.** 24 months imatinib, demarquation of metastases. **E.** 30 months imatinib, progressive tumor nodule (red circle). **F.** 30 months imatinib, progression liver metastases. **G.** 33 months, multiple progressive nodules (red circle). **H.** 33 months, progression of liver metastases.

**Figure 3:**
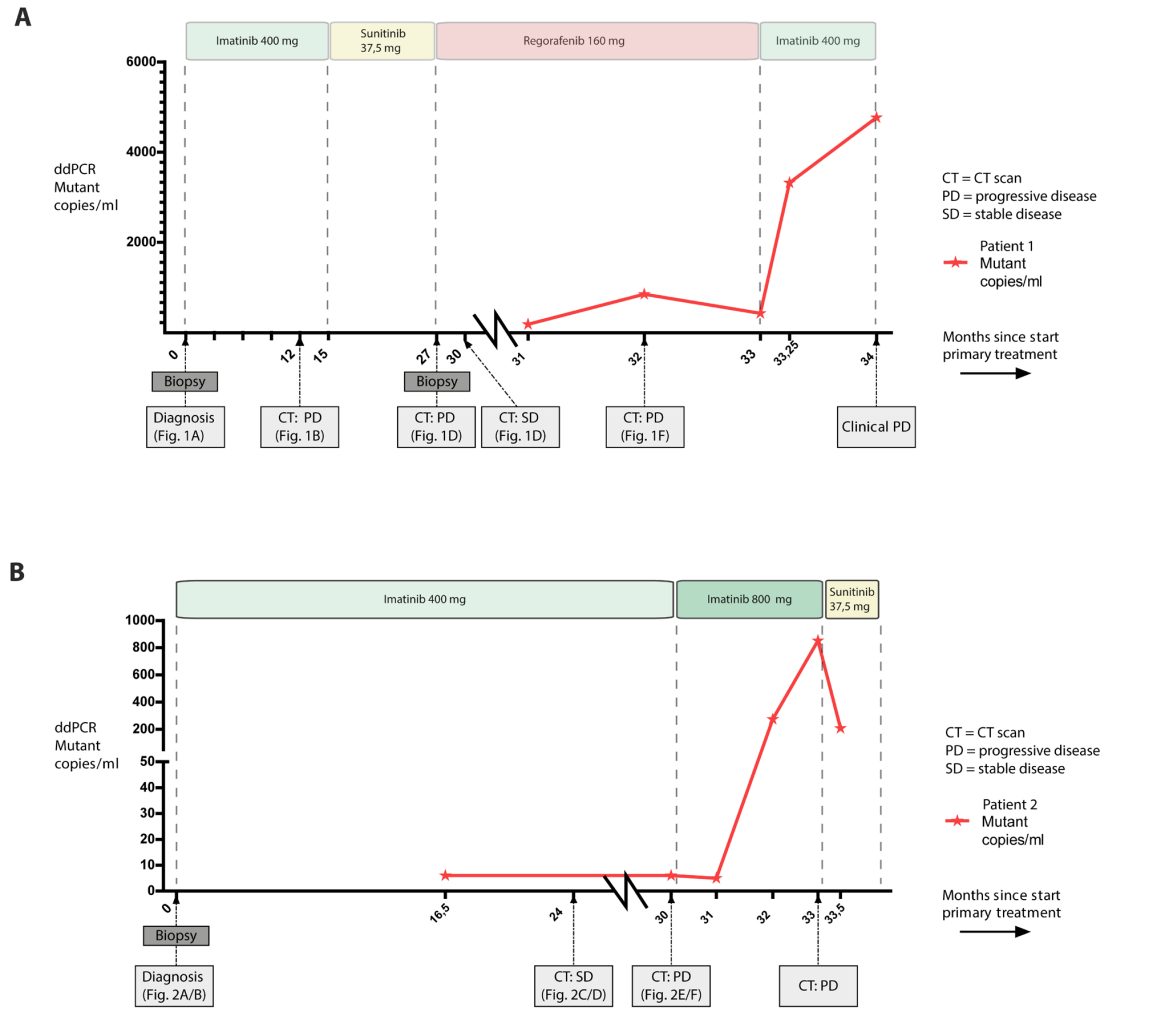
Serial plasma samples were analyzed during treatment The PDGFRα mutant copies/ml level as tested with ddPCR are shown. **A.** Patient 1 with the p.M844-S847del; c.2531_2542del variant. First plasma samples was collected 31 months after start of treatment, disease progression as determined with CT after 32 months corresponded with a rise in mutant copies/ml. Patient died 33 months after start of first treatment. **B.** Patient 2 with the p.I843_D846del; c.2527_2538del variant. First plasma sample was collected 16,5 months after start of treatment, disease progression at 30 months was not detected in plasma ctDNA, however after switch of therapy an increase in mutant copies/ml is detected corresponding with disease progression on CT at 32 months. After initiation of sunitinib, a decrease in mutant copies ml is seen. Patient died 36 months after initial diagnosis.

In total, this patient responded or had stable disease according to RECIST criteria for 32 months on several tyrosine kinase inhibitors. Mutation analysis performed on the progressive tumor nodule before start of regorafenib showed the same p.(M844_S847del) (c.2531_2542del) mutation in exon 18 of PDGFRα as detected in the primary tumor, whereas no additional mutations were found in exon 12 and 14 of PDGFRΑ nor in exon 9, 11, 13, 14, 17 of KIT (the average coverage is ∼2000 reads in tumor DNA with neoplastic content of 80%). Detection of the primary mutation in plasma reflected the clinical course of the disease in this patient.

### Clinical summary patient 2

A 76-year old woman was referred for analysis of abdominal pain. A CT scan showed a large abdominal tumor (diameter 25 cm) with multiple liver metastases (Figure 2A and 2B). Biopsy of a liver metastasis showed a CD117 positive tumor, characteristic for GIST. Mutational analysis showed no mutation in KIT, PDGFRα analysis was not performed at the time.

Treatment with imatinib 400 mg daily was initiated and after ten days she had clinical benefit. She tolerated the treatment well and a partial response was seen on the CT-scan performed every 3 months (Figure 2C and 2D). After 30 months of treatment progression of the primary tumor as well as the liver metastases was seen (Figure 2E and 2F). Additional mutation analysis was performed on the biopsy taken at diagnosis and revealed a PDGFRα exon 18 (NM_006206.5: c.2527_2538del; p.(I843_D846del)) mutation (the average coverage is ∼2500 reads in tumor DNA with neoplastic content of 95%). Treatment was continued with increased dosage of imatinib 400 mg twice daily and the patient tolerated this dosage, yet no clinical and radiological response was seen (progressive lesions on CT-scan after 3 months treatment, Figure 2G and 2H). Two months after the treatment was switched to sunitinib 37.5mg daily the patient continued to deteriorate and she died 36 months after the initial diagnosis. A tissue biopsy of a progressive lesion was not available to evaluate the secondary mutational status. Blood samples for ctDNA analysis (Figure 3B) were first drawn after 16,5 months of treatment with imatinib 400mg (6 mutant copies/ml). The mutant copies level remained stable until progressive disease was detected at the CT scan at 30 months. After initiation of imatinib 400mg twice daily an increase in mutant copies (5 to 275 /ml) was detected. The mutational level continued to rise to 852 mutant copies/ml corresponding with progressive disease on the CT scan performed at 33 months. After initiation of sunitinib treatment, a decrease in mutant copies (208 mutant copies/ml) was measured. Unfortunately, no further samples were available for analysis since the patient deceased after 2 months.

### Mutational analysis

For mutational analysis, DNA was extracted from formalin-fixed, paraffin-embedded (FFPE) tumor tissue using the Cobas DNA extraction kit (Roche, Basel, Switzerland). Next generation sequencing (NGS) analysis using the University Medical Center Groningen onco-panel on the Ion-Torrent platform (Thermo Fischer Scientific, Waltham, MA, USA) was performed. Torrent Suite Software was used to pre-process the raw data, and base calling, alignment, coverage analysis and variant calling was performed using SeqNext software (JSI medical systems GmbH) as reported previously [11]. According to international guidelines for clinical NGS panels [12], the minimal depth of coverage was set at 250 reads per tested amplicon. This to confidently identify also low frequency relevant variants in clinical tissues resulting from heterogeneity due to admixture of non-neoplastic cells, intratumoral variations (different clones) and viability of tumor cells. Relevant exons that are tested with this panel included KIT exons 8, 9, 11, 13, 14, 17 and PDGFRα exons 12, 14, 18 and BRAF codon 594, 599, 600 (www.moloncopath.nl).

### Computer 3D-modeling of the PDGFRα kinase domain

**Figure 4:**
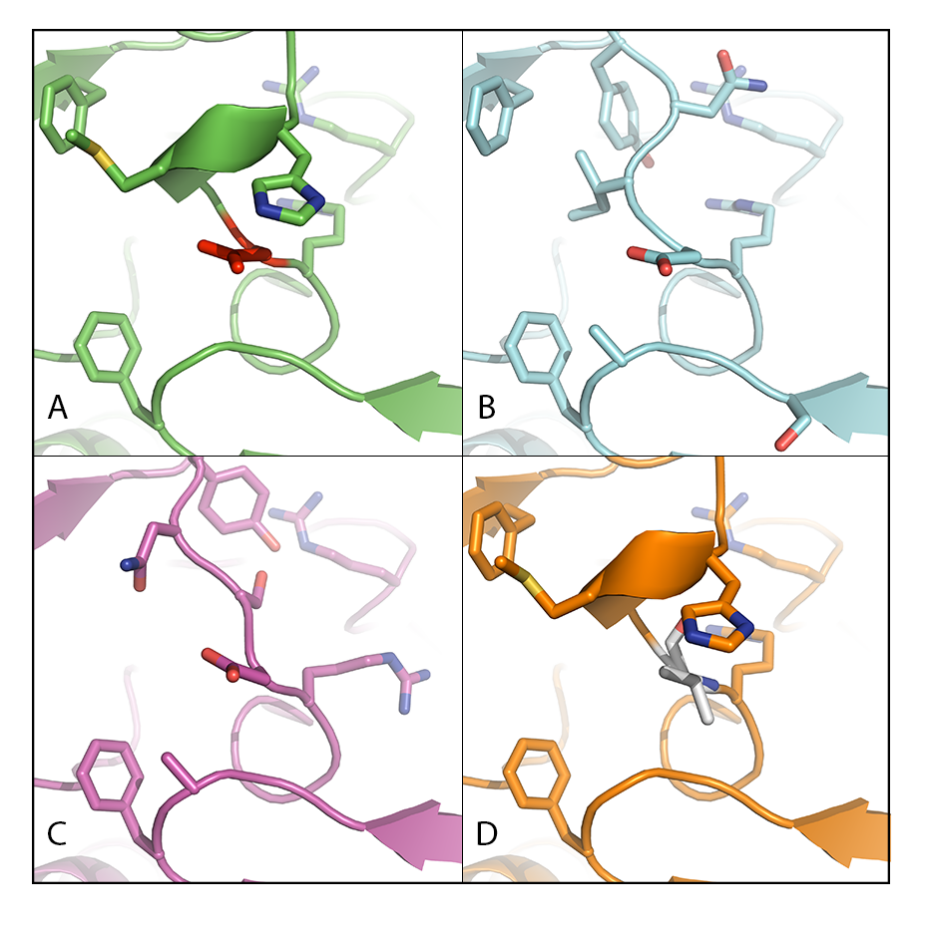
Residue 842 of human PDGFRα **A.** Residue D842 (in red) in the structure of wild type PDGFRα (pdb 5K5X). **B.** Residue D842 in the structure of wild type PDGFRα modeled with variant M844_S847del. **C.** Residue D842 in the structure of wild type PDGFRα modeled with the I843_D846del variant. **D.** Residue V842 (in grey) in the structure of wild type PDGFRα modeled with mutation D842V.

Computer 3D-modeling of the activation loop in the kinase domain of PDGFRα was recently reported to stabilize the kinase in the inactive state and to facilitate the binding of imatinib. The crystal structure with the D842V mutation suggested activation of the kinase and kinetic data confirmed an increased affinity for ATP both in agreement with the observed drug resistance in patients with the D842V mutation [13]. To evaluate the possible consequences of the I843_D846del and M844_S847del variants on the activation loop of PDGFRα, three 3D-models (PDGFRα-D842V, PDGFRα-M844_S847del and PDGFRα- I843_D846del) were built (Figure 4). The predicted orientation of D842, described to be essential for the auto-inhibited state of the tyrosine kinase domain [9], was conserved. The proposed hydrogen bond between D842 and H845(PDB; 4BKJ [14]) is almost certainly not formed, as the geometry of the interaction does not fall close to experimentally determined angular distributions [15]. Our 3D-modeling indicates that the PDGFRα M844_S847del and I843_D846del variants would play no role in activation.

In order to evaluate whether these 2 variants affect residues of PDGFRα that specifically interact with imatinib, the reported structure of the tyrosine kinase domain of DDR1 bound to imatinib (PDB; 4BKJ [14]) was used as template for homology modeling (35% sequence identity) [16]. Our model predicts that imatinib interacts with the same amino acids of DDR1 (E672, T701, V763, H764, D784) that are conserved in PDGFRα (E644, T674, V815, H816, D836). Considering that the PDGFRα M844_S847del and I843_D846del variants do not affect these 5 residues, it is highly likely that these variants will not directly affect binding of imatinib or sunitinib.

### Analysis of circulating tumor DNA

Blood samples were collected in EDTA tubes (vacutainer #367525, Becton Dickinson, Franklin Lakes, NJ, USA) and processed within 4 hours after vena puncture. Samples were processed and isolation of DNA were performed as described elsewhere [17]. Digital droplet PCR (ddPCR) primers and probes was in-house designed and ordered at IDT (Coralville, IA, USA). The primer sequences for patient 1 (p.M844_S847del; c.2531_2542del) were Fwd. 5’-CTCCTGGCACAAGGAAA-3’ (c.2473-c.2489) and Rev. 5’-GGACGTACACTGCCTTT-3’ (c.2554-c.2570) resulting in a PCR product of 98 base pairs. The sequence of probe I (FAM) was 5’-GCCAGAGACATCAACTATGTGTCG-3’ and probe II (HEX) 5’-CATGCATGATTCGAACTATGTGTCG-3’. For patient 2 (p.I843_D846del; c.2527_2538del) the primer sequences were Fwd. 5’-ATTGTGAAGATCTGTGACTTTG-3’ (position c.2491-c.2512) and Rev. 5’-AGTGAGGGAAGTGAGGA-3’ (position c.2568-c.2584) resulting in a PCR product of 94 base pairs. The sequence of probe I (FAM) was 5’-GCCAGAGACTCGAACTATGTGTCG-3’ and probe II (HEX) 5’-TGCATGATTCGAACTATGTGTCGAA-3’. Temperature gradient PCR of the primers and probes were performed to detect the optimal annealing temperature and resulted in an optimal PCR temperature of 55°C for both assays. The specific assays were validated on available tumor tissue. DdPCR was performed on a T100 Thermal Cycler (Bio-Rad, Hercules, CA, USA) and samples were transported to the QX200 Droplet Reader (Bio-Rad) for fluorescent measurement of FAM and HEX probes, data was analyzed with Quantasoft software version 1.6.6.

## REVIEW OF THE LITERATURE

### PDGFRα mutations and response on imatinib

**Figure 5:**
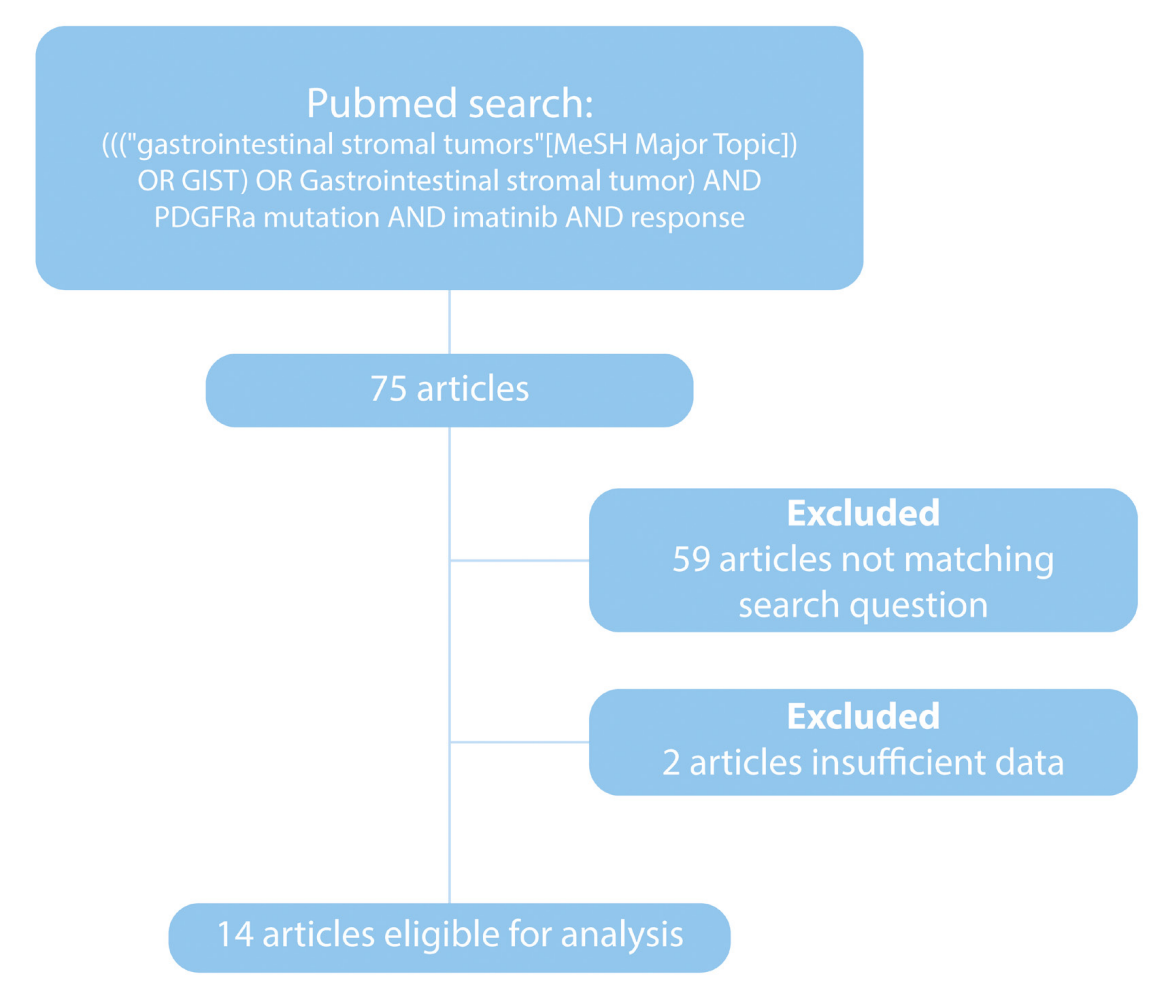
Flow-chart of search strategy In total 61 articles were excluded because of other study questions or insufficient data, resulting in 14 papers eligible for analysis.

PubMed was searched for articles concerning GIST patients with PDGFRα mutations and response to treatment with imatinib. Data of 14 papers and a total of 102 patients with PDGFRα exon 18 mutated GIST were retrieved by our search strategy (Figure 5).

Five of the fourteen papers describe data of progression free survival (PFS) or overall survival (OS) of patients with a PDGFRα mutated advanced GIST treated with imatinib (Table 1). A 10-fold increase in PFS is seen in patients with non-D842V mutated GIST compared with D842V mutated who were treated with imatinib. Recently, a series of 823 GIST patients including 13 patients with a PDGFRα exon 18 mutated GIST who were treated with first line imatinib is described [18]. The OS of patients with a D842V mutation was 25.2 months compared to 59.8 months for patients with a non-D842V PDGFRα mutated GIST (p=0.02). At least two other studies showed a better median OS in patients with a non-D842V PDGFRα mutation compared to patients with a D842V mutation [18, 19].

**Table 1:** Patient numbers and survival data of 14 papers found by the performed literature search

Author	Year	Patients (% PDGFRA)	PDGFRA exon 18 (D842V)	PFS non-D842V	PFS D842V	OS non-D842V	OS D842V
Yoo [18]	2015	823 (2%)	13 (9)	29.5 m	3.8 m	59.8 m	25.2 m
Cassier [20]	2012	58	49 (32)	28.5 m	2.8 m	NR (46 m fu)	14.7 m
Valadao [21]	2012	60 (5%)	3	*	*	*	*
Kang [22]	2012	290 (1%)	3 (2)	SD	PD	*	*
Yoon [23]	2012	88 (5%)	4 (3)	*	*	*	*
Dileo [24]	2011	2	1(0)	29 m	*	*	*
Kim [25]	2009	113 (1%)	1(0)	SD	*	*	*
Ryu [26]	2009	47 (0%)	0	*	*	*	*
Heinrich [27]	2008	746 (1%)	8 (4)	*	*	> 12 m (med 40,8)	9.7 m
Lim [28]	2008	12 (0%)	0	*	*	*	*
Gomes [29]	2008	78 (4%)	3 (2)	*	*	*	*
Debiec – Rychter [30]	2004	39 (5%)	2 (3)	*	*	*	*
Heinrich [31]	2003	127 (5%)	6 (3)	*	*	*	*
Heinrich [32]	2003	40 (28%)	11 (6)	*	*	*	*

NR = not reached, PD = progressive disease, SD = stable disease, m = month, fu = follow up

In COSMIC (catalogue of somatic mutations in cancer; www.cancer.sanger.ac.uk/cosmic) the p.M844_S847del mutation is described in eight patients in five studies. All patients were treated surgically and had no evidence of disease or recurrence afterwards, so no information was reported regarding the response on treatment (Table 2).

**Table 2:** Overview of the literature regarding p.M844_S847 PDGFRα deletion

Author	Patients	Location	Risk category	Clinical features	Response imatinib
Lasota [4]	1	-	-	Not reported	Not reported
Sihto [33]	1	Stomach	Intermediate	Not reported	Not reported
Chang [34]	1	Stomach	High	No recurrence (19m)	Not reported
Silva [35]	2	Stomach	Low	No recurrence*	Not reported
		Peritoneum	High	No recurrence*	Not reported
Kang [36]	3	Stomach	High	No recurrence*	Not reported
		Stomach	Very low	No recurrence*	Not reported
		Stomach	High	No recurrence*	Not reported

* Follow up time is not reported

A few patients with a GIST with a p.I843_D846del mutation have been described, although information regarding the response to TKI treatment is scarce [20, 37-47].

### Response on sunitinib as second line treatment

Two papers report on response to second-line treatment with sunitinib. In nine patients, no objective response was seen [18]. Patients with the D842V mutation tended to show poorer PFS than those with non-D842V PDGFRα mutations (median PFS 1.9 months for D842V mutant vs. 7.3 months for non-D842V PDGFRα mutations; P = 0.26). Another cohort consisted of 11 patients with a PDGFRα mutated GIST [20]. Of those patients, three had disease stabilization for more than 6 months. No significant difference could be shown between the different PDGFRα mutations (PFS 2.1 months for D842V mutant and 7.8 months for other mutations, P = 0.2489).

## DISCUSSION

Our two case patients with a non-D842V PDGFRα mutation responded or had prolonged periods of non-progressive disease to various tyrosine kinase inhibitors. Although patients with advanced disease and a PDGFRα mutation can respond to treatment with imatinib, the overall survival of these patients is worse than that of patients with a KIT mutation bearing GIST. This is reported in studies with advanced GIST patients where a median survival of 57 months for patients expressing KIT compared to a median overall survival of 23.7 months for patients with a PDGFRα mutation is reached [20, 48]. Imatinib resistance was reported most frequently in GIST patients with PDGFRα mutations and wild type GISTs. However, the PDGFRα mutations were not specified [49]. In contrast to the mutation distribution described in literature of 10% PDGFRα mutations in GIST, most articles report a much lower fraction of patients with PDGFRα mutations, suggesting underreporting.

To our knowledge, this is the first report regarding a p.M844_S847del variant in relation to TKI treatment. We show the clinical importance of mutation detection as patients with specific PDGFRα mutations respond well on imatinib treatment. The published reports that were found in the PubMed search provide limited PFS and OS data. However, patients with a non-D842V mutation have a favorable prognosis when treated with imatinib compared to patients with a D842V PDGFRα mutated GIST. A recent report showed a better overall survival in patients with non-D842V when treated with imatinib compared to patients with D842V mutated tumors [18]. Due to the latter it is of great importance to differentiate between those.

The frequency of recurrence after surgery is lower in patients with gastric versus non-gastric PDGFRα mutated GIST, similar to patients with other mutations [50]. In metastatic D842V mutated GIST the role of TKI is very limited and the prognosis is clearly far inferior to other mutated GISTs [51, 52]. Several new therapies are being investigated for patients with a PDGFRα (D842V) mutation [27, 53], however none of them are already available for daily clinical practice.

TKI sensitive mutations are mostly located near the imatinib resistant D842V domain. It has been implied that primary resistance to imatinib correlates specifically with substitution mutations that affect residue D842 of the kinase activation loop [9]. Modifications of this domain are interfering with a swinging movement of the activation loop. This movement is linked to a conformational shift of the ATP binding pocket from an “open” or active set-up to a “closed” or inactive set-up. Since imatinib is an ATP competitor and binds exclusively to the closed form of the kinase, substitutions of PDGFRα D842 reduce the accessibility of the ATP pocket and thereby give relative resistance to the drug [13]. An increased affinity for ATP of the mutated tyrosine kinase domain has also been reported to contribute to the acquired resistance to imatinib [54]. In contrast to D842V, our 3D-modeling of the kinase activation loop indicates that the M844_S847del and I843_D846del variants would play no role in activation. In addition, homology modeling predicted that imatinib interacts with 5 residues in PDGFRα (E644, T674, V815, H816, D836) conserved with those in DDR1. Because the PDGFRα M844_S847del and I843_D846del variants do not affect these 5 residues, it is highly likely that these variants will not directly affect binding of imatinib or sunitinib. In summary, our 3D-modeling analysis indicates that PDGFRα proteins harboring the M844_S847del and I843_D846del variants would not directly interfere in imatinib or sunitinib binding and would not affect activity. Consequently, both variants would still allow binding and inhibition of imatinib and sunitinib. This is in good agreement with the observed response to imatinib in these two patients. However, to assess sensitivity to tyrosine kinase inhibitors of these particular mutations, cell line-based drug sensitivity analysis would be of added value.

Secondary resistance usually occurs between 6-24 months after start of imatinib treatment. Secondary mutations as cause of TKI insensitivity have been found in patients with primary KIT mutations and rarely in patients with primary PDGFRα mutations [5]. Alternative pathways for secondary resistance are activation of other growth pathways and loss of the remaining wild-type PDGFRα and overexpression of PDGFRα or other tyrosine kinase receptors [55]. In patient 1, a biopsy at progression was taken. However, in addition to the M844_S847del variant in exon 18 of PDGFRα, no secondary treatment resistant mutations were detected with NGS.

The detection of mutations in circulating tumor DNA from cell free plasma (ctDNA) of patients with GIST has been reported before [56-60]. In this study we report on the correlation between clinical course/treatment response and the detection of the tumor-specific PDGFRA mutations in ctDNA of serially taken blood samples of these 2 patients with GIST, showing the use of mutation detection in ctDNA in plasma to monitor treatment response. In patient 1, the clinical course correlated well with the ctDNA level. However, in patient 2 the moment of progression on first line treatment was not detected in the ctDNA. According to the CT scan at 30 months (Figure 2E/F) there is one progressive nodule detected implying treatment resistance. Some lesions became larger, but have a cystic aspect what in GIST could match with treatment response. Since there is little active tumor tissue, we suggest the limited DNA shed of the progressive nodule in this phase is below the detection level of ddPCR. The following CT scan (Figure 2G/H) shows massive progression which is preceded by a detectable rise in mutant ctDNA copies. Further research has to reveal the clinical value of detectable mutant ctDNA copies.

In conclusion, the p.M844_S847del and p.I843_D846del mutations are rare but have clinical importance since these specific mutations are associated by a response to treatment with TKIs. This report emphasizes the importance of mutational analysis of tumors and is exemplary for the implementation of personalized medicine. Mutational analysis should be performed of each primary and resistant tumor to increase the knowledge of primary and secondary resistant mutations. Mutation detection in ctDNA to assess treatment response seems feasible. We suggest to register patients with very uncommon genetic aberrations in a prospective international database to understand the tumor biology and obtain more evidence to predict treatment response and eventually contributing to the development of new targeted therapies.
